# Toward Personalized Immunotherapeutic Drug Monitoring with Multiplexed Extended‐Gate Field‐Effect‐Transistor Biosensors

**DOI:** 10.1002/smsc.202400515

**Published:** 2025-02-03

**Authors:** Trang‐Anh Nguyen‐Le, Christin Neuber, Isli Cela, Željko Janićijević, Liliana Rodrigues Loureiro, Lydia Hoffmann, Anja Feldmann, Michael Bachmann, Larysa Baraban

**Affiliations:** ^1^ Institute of Radiopharmaceutical Cancer Research Helmholtz‐Zentrum Dresden‐Rossendorf e. V. (HZDR) 01328 Dresden Germany; ^2^ Else Kröner Fresenius Center for Digital Health Faculty of Medicine Carl Gustav Carus Technische Universität Dresden 01307 Dresden Germany

**Keywords:** biosensors, extended gate, field‐effect‐transistor, immunosensors, immunotherapies, point‐of‐care, precision medicine

## Abstract

The selection and optimization of therapies for cancer patients urgently need personalization. Portable point‐of‐care electronic biosensors emerge as a groundbreaking solution contributing to better decision‐making in precision oncology. In this study, the innovative use of extended‐gate field‐effect‐transistor (EG‐FET) biosensors is showcased for monitoring the concentration and pharmacokinetics of immunotherapeutic drugs in vivo. Complementary positron emission tomography and radioactivity biodistribution studies in mice validate the EG‐FET measurements. Herein, a novel indirect assay format is also introduced for detecting target modules (TMs) in an adapter chimeric antigen receptor T‐cell therapy model, effectively addressing the current limitations of potentiometric measurements. In pharmacokinetic evaluations, the EG‐FET biosensor performance aligns with standard radioactivity measurements, revealing the distinct lifespans of small‐sized single‐chain‐fragment‐variable‐derived TMs (15 min) and larger IgG4‐derived TMs (14 h). Advantageously, the EG‐FET sensors exhibit exceptional sensitivity and fulfill the requirements for immunotherapeutic drug monitoring without complex radioactive labeling, which is indispensable. In these promising findings, the exploration of next‐generation electronic biosensors as therapeutic monitoring tools is advocated for. With their cost, size, and response time advantages, these biosensors hold immense potential for advancing personalized oncology, transcending the conventional diagnostic roles typically highlighted in the literature.

## Graphical abstract

In this study, a proof of concept is presented using an extended‐gate field‐effect‐transistor biosensing platform for monitoring immunotherapeutic drugs in vivo. Using varying sizes of immunotherapeutic drugs, the biosensor surpasses the sensitivity of standard methods, reducing the need for radioactive labeling in real‐time sensing. The innovation paves the way toward cost‐effective and personalized cancer therapy monitoring for improved patient outcomes.

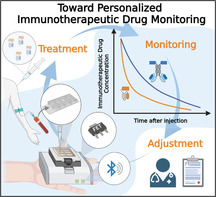

## 1 Introduction

**Figure 1:**
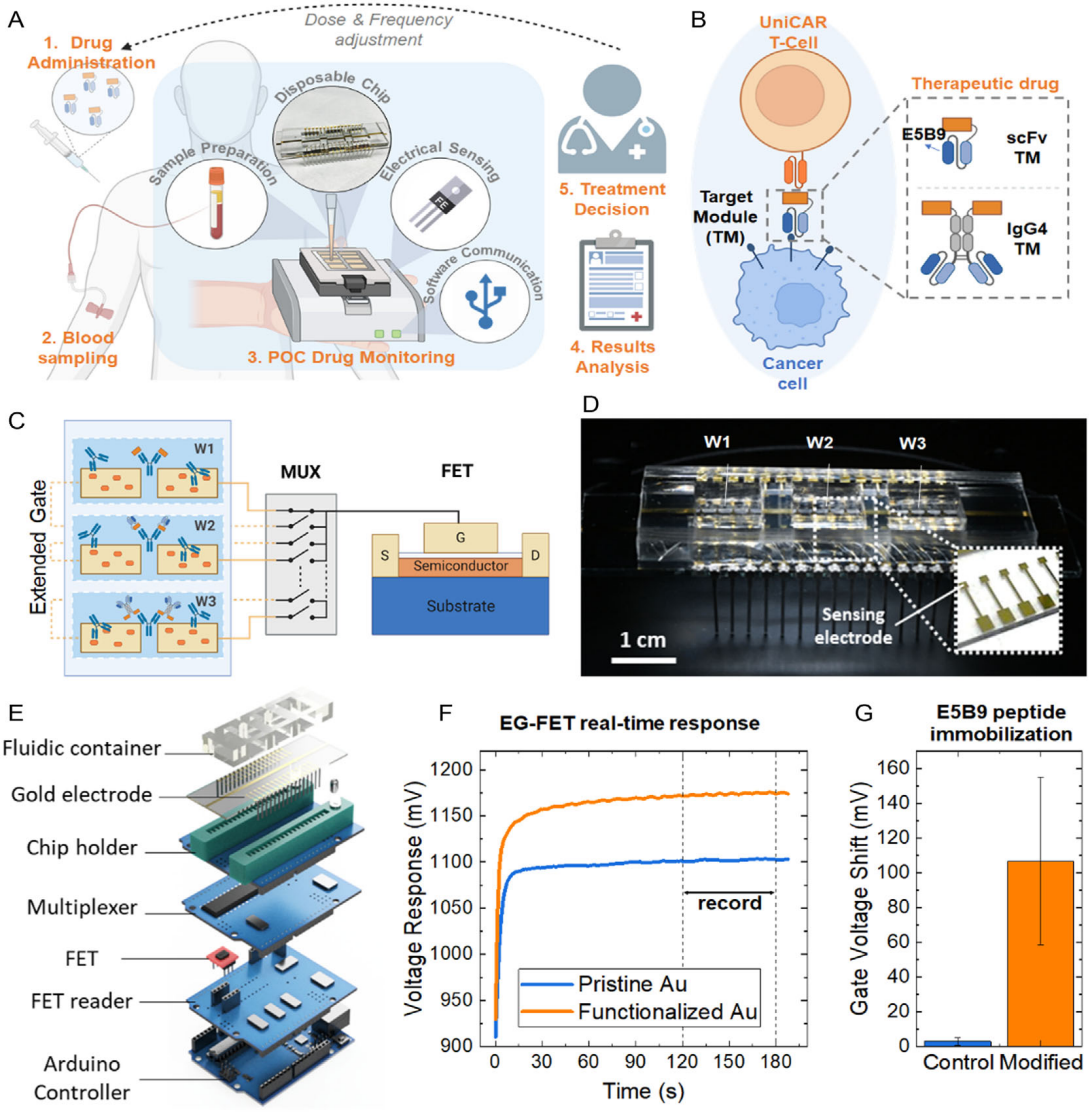
A) Schematic representation of the proposed approach for optimizing immuno‐oncological treatment with the application of a point‐of‐care (POC) drug monitoring device. B) Illustration of the UniCAR system in cancer immunotherapy. UniCAR T‐cells consist of an extracellular single‐chain fragment variable (scFv) targeting the peptide epitope E5B9. By adding an intermediary TM consisting of a unit that recognizes tumor‐associated antigens and the epitope E5B9 that interacts with UniCARs, redirecting UniCAR T‐cells to attack tumor cells is made possible. C) Illustration of the concept of our EG‐FET biosensing platform. W1, W2, and W3 represent three different testing wells enabling the study of different target reagents simultaneously. D) Assembly of the EG chip for sensing. A PDMS‐based fluidic container is attached to the chip to localize the liquid bioanalytes in wells W1, W2, and W3. The gold electrodes are fixed to the metallic pins for electrical connection. Inset: an exemplary photograph of our in‐house fabricated EG gold electrodes. The sensing surface is a square‐shaped gold area (1 × 1 mm). E) Illustration of the construction of our EG‐FET platform. The sensing chip, covered by the fluidic container, is mounted on a zero‐insertion‐force (ZIF) connector and connected to a multiplexer that performs the switching between measuring electrodes. For consistency, a single commercial FET is used to transduce the potential change of all electrodes. The platform is controlled by an Arduino Uno board. The data is displayed and processed using custom MATLAB scripts. F) Real‐time response of an electrode before and after surface functionalization with E5B9 peptides. Potentiometric recording of electrode response was carried out for 3 min in all measurements. Only steady‐state data recorded in the period between 120 and 180 s were used for calculation and comparison. G) Comparison of voltage shifts between control (non‐modified) and modified electrodes after surface functionalization with E5B9 peptides. The error bars indicate the standard deviations (SDs) of *n* = 12 electrodes.

Anticancer treatments require a high degree of personalization to effectively address each patient's unique characteristics.^[^ ^1^, ^2^ ^]^ Fortunately, the advent of miniaturized sensing technology offers a new frontier in achieving even greater levels of customization, enhancing treatment precision and efficacy in numerous ways.^[^ ^3^, ^4^ ^]^ In the landscape of contemporary cancer treatment, immunotherapy has emerged as a revolutionary approach, employing immunotherapeutic agents, such as monoclonal antibodies, checkpoint inhibitors, cancer vaccines, and chimeric antigen receptor (CAR) T‐cell therapy, to assist the body's immune system in its battle against cancer.^[^ ^5^, ^6^ ^]^ This transformative approach has ushered in a new era in oncology, marked by enhanced clinical efficacy and higher survival rates. However, the emergence of immunotherapy has catalyzed a paradigm shift in the traditional drug development framework.^[^ ^7^ ^]^ Unlike conventional cancer drugs, immunotherapeutic agents are engineered to activate and harness the body's immune response rather than exert direct cytotoxic activity on tumors. Consequently, the conventional criteria for drug dose determination, often focusing on exposure levels, have become less relevant in immunotherapy. Instead, the prominence of immune‐related adverse effects, influenced more by individual patient immunity and susceptibility, challenges the conventional dosing models. The complexity of navigating the landscape of cancer treatment using immunotherapy underscores the enduring relevance of therapeutic drug monitoring for individualized dosing strategies (**Figure** **1**A). Drug monitoring yields twofold benefits in practice. First, it optimizes treatment efficacy and safety through a nuanced comprehension of individual pharmacodynamics and pharmacokinetics of certain immunotherapeutic agents.^[^ ^8^, ^9^, ^10^ ^]^ Second, it facilitates cost‐effectiveness in utilizing high‐cost immunotherapy agents by ensuring judicious resource allocation.^[^ ^11^, ^12^ ^]^ Although enzyme‐linked immunosorbent assay (ELISA), positron emission tomography (PET),^[^ ^13^ ^]^ and magnetic resonance imaging (MRI)^[^ ^14^ ^]^ have been employed in preclinical and clinical studies to monitor active drugs, their inherent limitations hinder practical integration into routine clinical practice at later stages. Specifically, the low sensitivity of ELISA and the high‐cost and time‐consuming nature, along with the requirement for labeled tracers in PET and MRI, underscore the urgent need to develop new, more adaptable tools, suitable for personalized analysis. Advancements in drug monitoring tools and techniques are essential for the effective navigation of the continuously evolving landscape of cancer immunotherapy and one of the prerequisites for entering the era of personalized medicine (Figure 1A).

To address the limitations of current monitoring tools, there is a growing shift toward portable sensing technologies for personalized therapy optimization.^[^ ^15^, ^16^, ^17^ ^]^ The field‐effect‐transistor (FET)‐based biosensors^[^ ^18^, ^19^ ^]^ have emerged as miniature, rapid, and highly sensitive tools with demonstrated success in various sensing applications, exemplified by their use in detecting clinically relevant biomolecules like stress hormone,^[^ ^20^, ^21^, ^22^, ^23^ ^]^ cardiac troponin I,^[^ ^24^ ^]^ and prostate cancer biomarkers.^[^ ^25^ ^]^ Previously, our research group showcased the remarkable sensitivity of nanowire FET biosensors and their applicability in immunotherapeutic drug development and screening.^[^ ^26^ ^]^ Nevertheless, the micro‐ and nano‐fabrication processes involved in constructing these sensors necessitate access to complex facilities and highly specialized expertise. Furthermore, these processes can have inherently limited scalability, increasing the processing times and production costs. Recently, the extended‐gate FET (EG‐FET) approach, inspired by the traditional electrolyte‐gated FET biosensors but with an electrical connection between the gate of a conventional FET and a separate sensing electrode, has gained recognition through successful demonstrations by multiple research groups.^[^ ^27^ ^]^ The physical separation of the sensing interface and the transducer in EG‐FET sensors offers greater flexibility in sensor design and facilitates effective FET packaging with the other components of the reusable electronic measurement system. This innovation overcomes longstanding limitations of electrolyte‐gated FET sensors, including issues like ion penetration into the oxide layer, sensitivity to environmental factors, and demanding fabrication requirements. The versatility of the EG‐FET sensor design and its impressive track record in detecting pivotal biomolecules position it as a promising advancement in biosensor technology.

Unlike previous studies, here we investigate the potential of the EG‐FET biosensor as a tool for therapeutic drug monitoring in vivo. We use a “Target Module” (TM), a key component of the universal CAR (UniCAR) T‐cell system,^[^ ^28^, ^29^ ^]^ as a model immunotherapeutic agent. The concept of UniCAR T‐cell technology with the use of TM is illustrated in Figure 1B and S1, Supporting Information, respectively. The presence of the TM is essential for bridging the interaction between CAR T‐cells and the tumor, thereby initiating the cytotoxic activity of T‐cells. These modular CAR variants not only enhance functionality but also facilitate the management of potential side effects of adaptor CAR T‐cells, by enabling precise regulation of the TM concentration in patients, as already confirmed in the first clinical Phase 1 trials for the UniCAR system (NCT04633148, NCT04230265).^[^ ^30^, ^31^ ^]^ As TMs can be created in various formats ranging from small modules to larger Ig‐based modules, their respective size determines their pharmacokinetic behavior. Bearing in mind that low concentrations of TMs, below the limit of detection (LOD) of currently available analytical systems, are sufficient for activation of UniCAR T‐cells, easy, fast, and highly sensitive tools are indispensable to monitor the TMs in blood for precise steering of therapy efficacy and potential side effects in patients.

Here, we show a versatile routine that can be effectively applied to all types of therapeutic agents, where TMs are utilized to initiate the therapy. In this endeavor, we present an indirect assay format designed to enhance the sensitivity of EG‐FET biosensors. Building upon the EG‐FET biosensing system,^[^ ^32^ ^]^ we have employed this strategy to detect TMs of varying sizes in serum samples. The TMs used in this study are strategically engineered to target the fibroblast activation protein (FAP), a cell surface protein overexpressed in a broad spectrum of cancers^[^ ^33^ ^]^ and, particularly, in cancer‐associated stromal cells, such as cancer‐associated fibroblasts, within the tumor microenvironment. Within the scope of this study, the established small‐sized scFv TM (32 kDa) and a large‐sized immunoglobulin G4 (IgG4) TM (112 kDa) targeting FAP were used^[^ ^34^ ^]^ as also illustrated in Figure 1B and S1, Supporting Information, respectively. In pursuit of comprehensive insights, we investigate EG‐FET sensing signals generated by the interactions of both TMs in serum and study their pharmacokinetics in murine models. In parallel, we perform other gold‐standard techniques such as optical ELISA, PET, and radioactivity biodistribution studies to benchmark our technique. Preliminary results showed that our EG‐FET sensors are 1000 times more sensitive than ELISA, making them suitable for measuring injected UniCAR immunotherapeutic drugs below the nM range while eliminating the need for radioactive labels required for PET and radioactivity measurements. This research aims to explore the application of FET‐based biosensors, particularly in therapeutic monitoring and evaluation, thereby transcending their conventional role in early diagnostics and focusing on personalized medicine.

## 2 Results and Discussion

### 2.1 EG‐FET Biosensing Platform

The operational concept of an EG‐FET biosensor is illustrated in Figure 1C. In this study, we employ a custom‐designed EG‐FET system.^[^ ^32^ ^]^ The fully disposable sensing chip, as illustrated in Figure 1D and S2, Supporting Information, features a simple and practical design that enables mass production. It is designed for plug‐and‐measure functionality, ensuring ease of use, reliability, and cost‐effectiveness. This approach makes the chip highly suitable for scalable applications and consistent performance in clinical and research settings. We have engineered a multiplexed readout platform that operates using a single commercial FET transducer for potentiometric measurements. Figure 1E presents all the key components of our EG‐FET biosensor system, including a multiplexer, responsible for establishing electrical connections between specific contact pads on the sensing chip and the gate terminal of a commercial n‐channel enhancement mode metal–oxide–semiconductor FET on the FET reader module. This configuration allows for seamless switching between different sensing electrodes during the readout process. The FET reader operates in constant charge mode with predefined values of the drain‐to‐source voltage (*V*_DS_) and drain current (*I*_D_) of the FET transducer. This readout configuration ensures that potential variations at the gate electrode are equivalent to the potential variations at the source terminal of the FET. Therefore, we can record an output signal obtained by measuring the potential difference between the source terminal of the FET and the reference electrode.

The surface of the gold electrodes is chemically treated to attach the E5B9 peptide via its relevant functional groups, allowing the execution of the indirect assay.^[^ ^26^ ^]^ Bovine serum albumin (BSA) is used to block the remaining surface. The entire functionalization process is monitored *in situ* using both electrochemical impedance spectroscopy (Figure S3, Supporting Information) and potentiometric EG‐FET‐based measurements (Figure S4, Supporting Information), which yield congruent results. In our previous work, this immobilization approach demonstrated stable performance after up to 72 h of storage.^[^ ^32^ ^]^ Figure 1F depicts a real‐time voltage response of an electrode before and after E5B9 peptide functionalization. Notably, our multiplexer facilitates the simultaneous reading of data from 32 electrodes and the concurrent incubation of multiple analytes, e.g., serum samples from different time points during pharmacokinetics studies (see Figure 1C) in different polydimethylsiloxane (PDMS) wells, significantly enhancing the measurement throughput. The primary parameter under examination is the voltage shift, defined as the voltage response difference between the initial and conditioned states of an individual electrode. Figure 1G illustrates the voltage shift for two groups of electrodes: pristine gold electrodes preserved in a sealed container (blue) and gold electrodes functionalized with E5B9 peptides (orange). A significant difference between the two groups (*P* < 0.0001) indicates a change in the surface charge attributed to the presence of E5B9 peptides on the sensing surface.

### 2.2 Universal Indirect Detection Approach

**Figure 2:**
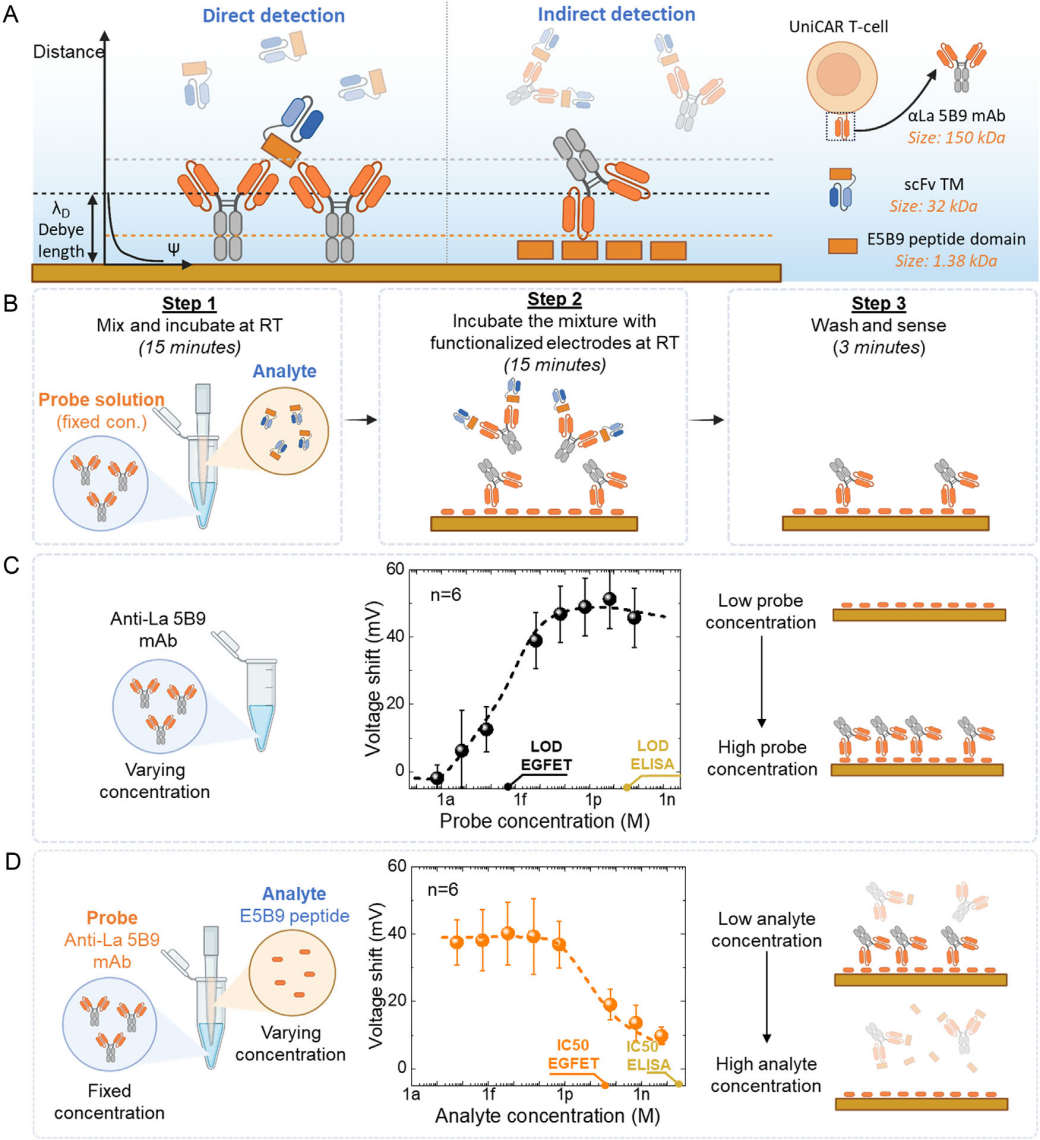
Illustration of the indirect detection assay on the EG‐FET platform. A) Schematic illustration of the electrical double layer with designated Debye length in direct and indirect approaches for TM detection (dimensions are not to scale). B) Illustration of a step‐by‐step procedure for conducting indirect TM detection with our EG‐FET platform. C) EG‐FET calibration results for varying concentrations of probe molecules (αLa 5B9 mAb). D) EG‐FET calibration results for varying concentrations of analyte molecules (E5B9 peptides) at a fixed concentration of probe molecules αLa 5B9 mAb (666 fM). All error bars indicate the standard deviation of *n* = 6 electrodes. LOD: limit of detection. Illustrations are partially created by Biorender.com.

To achieve immunotherapeutic TM detection, we used anti‐La 5B9 monoclonal antibodies (αLa 5B9 mAb). As mentioned earlier, the αLa 5B9 mAb specifically targets the E5B9 epitope found in all UniCAR TMs, allowing their broad application. The αLa 5B9 mAb is a full‐size IgG antibody with a molecular weight of ≈150 kDa. However, in the context of FET‐based biosensors, the substantial size of full IgG antibodies poses a limitation due to sensitivity challenges caused by the screening of electric fields by mobile ions in biological samples.^[^ ^35^ ^]^ Researchers often assess this issue by employing a capacitive model^[^ ^36^ ^]^ based on the Poisson–Boltzmann formulation to estimate the Debye length (*λ*_D_), a key parameter that characterizes the extent of screening at the electrode–electrolyte interface. Effective potentiometric measurements are typically possible only within the range of distances from the electrode surface that are smaller than the *λ*_D_. When measuring in solution with ionic strength close to physiological conditions, *λ*_D_ is lower than 1 nm, while a full‐size antibody typically measures in the range of 10–15 nm, rendering direct detection challenging beyond this distance^[^ ^35^ ^]^ (refer to **Figure** **2**A). Various solutions have been proposed to overcome the Debye length limitation, including dilution of the measurement medium;^[^ ^37^ ^]^ biomolecular engineering to reduce bioreceptor size;^[^ ^38^, ^39^ ^]^ utilization of Debye volume to reduce screening, such as coating the sensing layer with a large polymer^[^ ^40^ ^]^ or employing nanostructures;^[^ ^41^, ^42^, ^43^, ^44^ ^]^ implementing an ion‐permeable layer;^[^ ^45^ ^]^ analyte labeling^[^ ^32^ ^]^ and nonequilibrium double‐layer measurements.^[^ ^46^ ^]^ In this study, we present an indirect detection approach that leverages existing materials, obviating the need for reengineering the measurement system or the production of suitable smaller biomolecules.

In the indirect detection method, the sensing surface is modified with E5B9 peptides (Figure 2A), which are significantly smaller in size (1.38 kDa) than αLa 5B9 mAb (150 kDa). Simultaneously, known concentrations of αLa 5B9 mAb are prepared and mixed with the analyte‐containing solution. E5B9‐containing TMs effectively occupy and block the available binding sites on the αLa 5B9 mAb, preventing them from binding to the sensing surface and subsequently generating a sensing signal. In samples with low TM concentration, not all the binding sites on the αLa 5B9 mAb are saturated, resulting in a higher binding to the E5B9 peptide on the sensing surface and, therefore, a higher signal. The stepwise process of the indirect detection procedure is visually depicted in Figure 2B.

We validate the concept through a parallel assessment involving the gold‐standard ELISA method (Note S1, Supporting Information) and measurements conducted using our EG‐FET system. First, we evaluate the binding affinity of E5B9 peptides and αLa 5B9 mAb, as well as the functionality of our EG‐FET system by detecting the direct binding of the probe αLa 5B9 to the E5B9 peptide on the sensing surface. The biosensing experiments followed the procedures outlined in the Experimental Section. In summary, we incubated 0.6 mL of αLa 5B9 mAb, which had been appropriately diluted in a 10 mM phosphate‐buffered saline (PBS) solution (pH 7.4), prepared at predetermined concentrations ranging from 1 aM to 100 pM, for 15 min in the PDMS reservoir. The measurement of voltage response after 15 min of incubation with PBS was considered as the baseline for calculating the voltage shift. The calibration curve for the direct detection of the probe αLa 5B9 mAb is shown in Figure 2C. In both approaches, the peptide and antibody exhibit good affinity, with a calculated equilibrium dissociation constant (*K*_D_) of 0.19 fM determined via our EG‐FET calibration curve using Michaelis–Menten fitting (see ref. [26]). The model was chosen for its simplicity and suitability for comparing affinities within the same system. As anticipated, the EG‐FET sensing surface modified with the E5B9 peptide demonstrates significantly higher sensitivity (11.8 mV dec^−1^) than with the full‐size IgG antibody without amplification labeling (4.4 mV dec^−1^).^[^ ^32^ ^]^ Additionally, our EG‐FET platform achieves a remarkable *≈*80 times lower LOD (0.65 fM) compared to the gold‐standard optical ELISA‐based measurements (53 pM) for αLa 5B9 mAb detection (Figure S5A, Supporting Information).

Subsequently, we conducted tests to validate the indirect detection concept using the E5B9 peptide as the analyte, given its reliable affinity to αLa 5B9 mAb and its universal applicability for TMs. In this configuration, αLa 5B9 mAbs served as probe molecules, consistently diluted in PBS at a fixed concentration of 666 fM (equivalent to 100 pg mL^−1^). Various concentrations of solutions containing E5B9 peptide, ranging from 100 aM to 10 nM, were also prepared in PBS. A mixture of the probe and analyte solution (1:1 vol/vol ratio) was incubated for 15 min before application to the sensing surface. This mixture was then introduced into the EG‐FET system for a 15 min incubation, followed by thorough washing. The subsequent measurement recorded the voltage shift compared to the baseline established with pure PBS. The calibration outcome for the E5B9 peptide in the indirect detection format is visually presented in Figure 2D. Both ELISA (Note S1, Figure S5, Supporting Information) and potentiometric EG‐FET measurements consistently indicated an inverse relationship between the sensing signal in indirect detection and the analyte concentration, where the signal change correlated with the concentrations of the used probe (αLa 5B9 mAb). Because of the competitive nature of the assay, an increase in the number of TMs leads to a decrease in the number of αLa 5B9 mAbs that bind to the sensing surface, resulting in a lower signal. Since each probe molecule theoretically possesses two accessible binding sites for analytes, ideally, there is no binding to the sensing surface, and thus, there is no signal at analyte concentrations two times higher than that of the probe. Nevertheless, experimental data showed that doubled probe concentration (1.33 pM) marks a decrease in the sensor signal rather than its complete absence, which might indicate limited binding efficiency in solution. A similar phenomenon is observed in ELISA tests (Figure S5B, Supporting Information) despite the much longer incubation time applied. The half‐maximal inhibitory concentration (IC50), a benchmarking parameter for assessing the sensitivity of competitive immunoassays,^[^ ^47^ ^]^ reveals a nearly 1000‐fold enhancement in sensitivity for EG‐FET (IC50 = 28 pM) compared to the most favorable outcome achieved with ELISA (IC50 = 52.3 nM). This substantial improvement underscores the remarkable performance of the EG‐FET biosensing system in detecting and quantifying target molecules. These results unequivocally showcase the potential and efficacy of employing an indirect assay format with the EG‐FET biosensing system for the semiquantitative assessment of target molecules in diverse samples.

### 2.3 Detection of TMs in Serum Sample

**Figure 3:**
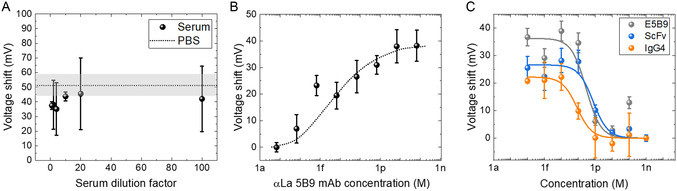
EG‐FET sensing response in serum samples. A) Study of the contribution of serum in EG‐FET biosensing. Voltage shift in response to the spiking of 6 nM αLa 5B9 at different serum dilution factors. The dashed line represents the voltage shift of PBS spiked with 10 ng mL^−1^ (6 nM) αLa 5B9. The shaded area indicates the standard deviation for the measurements in PBS (*n* = 6). B) Calibration results for αLa 5B9 mAb probe molecules in 100× diluted serum. C) Calibration results for indirect assays on IgG4, scFv TM, and E5B9 peptide analytes spiked in 100× diluted serum with fixed probe concentration of 100 pg mL^−1^ (666 fM). All error bars indicate the standard deviations of *n* = 6 electrodes.

To further assess the applicability of our approach, we employed it for the detection of TMs in blood serum samples rather than PBS, which presents a more relevant scenario for clinical applications. Serum, a product of blood purification after removing blood cells and clotting factors, still contains various proteins, antigens and antibodies, as well as residual immunotherapeutic agents like TMs in the case of UniCAR therapy. Hence, there is a potential to monitor the concentration of TMs in serum during treatment, improving clinical decision‐making and providing benefits for pharmacokinetic studies in drug development. **Figure** **3**A displays the sensing signals obtained through our EG‐FET potentiometric measurements for detecting the probe molecule αLa 5B9 mAb at a fixed concentration of 6 nM in serum at varying dilution ratios in PBS. It is essential to emphasize that the voltage shift is determined in comparison to the baseline for the identical serum dilution factor (without the introduction of probe molecules). On average, the signal amplitude in serum is lower than in PBS, albeit not significantly (error bars overlapped). Furthermore, the dilution factor does not notably impact the signal level. However, due to the limited sample volume available from each blood withdrawal, we opted to work with a 100× diluted serum in PBS. The calibration curve for the probe molecules in the 100× diluted serum is presented in Figure 3B. This curve indicates a reduction in sensitivity to a half of that observed in PBS, with a sensitivity of 6.00 mV dec^−1^, while slightly reducing affinity to the E5B9 peptide immobilized on the sensing surface within the sub‐femtomolar dissociation constant range (*K*_D_ = 0.19 fM in PBS and *K*_D_ = 0.26 fM in serum). Furthermore, the LOD remains consistently within the range of femtomolar concentrations.

In the subsequent phase, we conducted indirect assay experiments using mice serum with three distinct analytes: E5B9 peptides, small‐sized scFv TMs, and large‐sized IgG4 TMs, as depicted in Figure S1B, Supporting Information. Throughout this experiment, the 100× diluted serum was enriched with analytes at concentrations ranging from 0.1 fM to 1 nM, while probe molecules, αLa 5B9 mAbs, were consistently prepared in PBS at a fixed concentration of 100 pg mL^−1^ (666 fM). The analyte‐containing diluted serum and probe solution were mixed in a 1:1 volume ratio and incubated for 15 min. The calibration curves for the three analytes are shown in Figure 3C. Notably, the calibration curve for the E5B9 peptide in a diluted serum exhibits a leftward shift compared to the calibration curve obtained in pure PBS (see Figure 2D), reducing the IC50 in diluted serum to 283 fM. This observation suggests an increase of interference from the complex composition of blood serum, which could lead to the blocking of binding sites on the sensor and of αLa 5B9 mAb. The scFv TM, comprising a single E5B9 epitope, behaves in a remarkably similar manner with slightly higher IC50 = 750 fM. A more intricate situation emerges with IgG4 TMs since each molecule encompasses two E5B9 epitopes attached to the heavy chains (Figure S1, Supporting Information). With two E5B9 epitopes, IgG4 TMs saturate the probe molecules (αLa 5B9 mAb) at a lower concentration. Consequently, the IgG4 TM exhibits a distinct calibration curve compared to the other two analytes. The signal began to level off at an analyte concentration lower than the used probe concentration, leading to a lower IC50 of 84 fM. The “ON” signal levels (maximum sensing response) of the three analyte molecules exhibit slight variations, possibly due to their distinct structure and charge distribution. Different TMs occupy binding sites of probe αLa 5B9 mAb, resulting in the formation of different complexes with varying overall charges inducing different potential shifts, and thus different “ON” levels.

These findings underscore the significant promise of our potentiometric biosensing system for detecting TMs in clinical blood serum samples. Notably, in previous clinical trials, TM doses administered to patients fell within the range from 0.5 to 1 mg for each treatment,^[^ ^30^ ^]^ equivalent to concentrations from 3 to 7 nM.^[^ ^31^ ^]^ Furthermore, studies have shown that tumor lysis can occur at TM concentrations as low as a few picomolar (pM),^[^ ^48^, ^49^ ^]^ levels where conventional methods like ELISA fail to detect TMs due to their higher LODs. While alternative methods, such as radioactive labeling, can achieve lower LODs, they present challenges, including procedural complexity and the need for specialized facilities. The LOD achieved in this study (fM range) demonstrates sufficient sensitivity of the EG‐FET biosensing system for clinical detection even with samples diluted by a factor of 100. Excellent sensitivity and the ability to detect ultralow analyte concentrations within complex serum samples strongly support the practical utility of our biosensing approach for clinical applications, particularly in the context of UniCAR T‐cell therapy.

### 2.4 Pharmacokinetics Study of TMs in Animal Models

**Figure 4:**
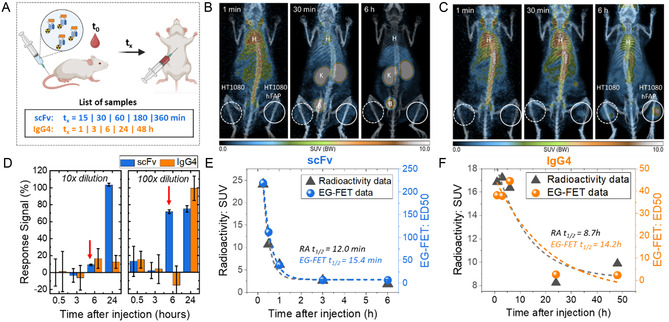
A) Concept figure of pharmacokinetic study in mice (partially created by Biorender.com). PET images present biodistribution of B) scFv and C) IgG4 TMs over time after i.v. injection. The color scale represents the standardized uptake value (SUV) (H: heart, K: kidney, B: bladder). D) EG‐FET sensor response recorded for samples at different time points and dilution factors. Red arrows indicate the onset of signal shift. Comparison of processed data acquired from radioactivity and EG‐FET measurements for E) scFv and F) IgG4 TMs. Dashed lines designate a single‐phase exponential decay fitting of each dataset. FAP‐negative (HT1080) and hFAP‐positive tumors (HT1080 hFAP) are enclosed by the dashed circle outlines on the left and solid circle outlines on the right within panels (B) and (C), respectively.

To further assess the potential of our technology, an in vivo experiment was conducted to investigate the pharmacokinetics of scFv and IgG4 TMs using radioactivity‐based biodistribution experiments and EG‐FET immunosensors. Both TMs were functionalized with the chelator 1,4,7‐triazacyclononane, 1‐glutaric acid‐4,7 acetic acid (NODAGA) and radiolabeled with the positron emitter copper‐64 (Note S2, Supporting Information). Given the structural and size differences between the two TM formats, distinct pharmacokinetic profiles are confirmed by PET (Note S3, Supporting Information, **Figure** **4**B,C) and these results are in line with the previous findings.^[^ ^34^ ^]^ The 32 kDa scFv TM demonstrates rapid kidney uptake and excretion through the bladder (Figure 4B). In contrast, the larger IgG4 TM, with a size of 112 kDa, cannot be excreted via urine (Figure 4C), resulting in extended blood circulation. Using high‐temporal‐resolution PET measurements, enabling a two‐phase decay (fast distribution and slow elimination phase) fitting of radioactivity concentration in blood versus time, we ascertain a total half‐life in the blood of 11 min for scFv and 16 h for IgG4 (Figure S10, Supporting Information). However, due to the limited spatial resolution of PET and the related partial volume effect,^[^ ^50^, ^51^ ^]^ radioactivity quantification in small lesions like murine blood vessels becomes difficult with PET.

Therefore, the pharmacokinetics of TMs was additionally investigated by in vivo measurements of radioactivity concentration in organs of interest (biodistribution, Note S4, Supporting Information). Following intravenous (i.v.) injection, mice were sacrificed at specific time points post‐injection (Figure 4A) to enable in vivo measurements of TMs, e.g., in blood serum through radioactivity concentration assessments (Note S4, Supporting Information). Data from biodistribution experiments (Figure S11A,B, Supporting Information) confirm PET results, and allow for the determination of TM pharmacokinetics in blood versus serum (Figure S11C,D, Supporting Information). Finally, serum samples were analyzed by EG‐FET immunosensors. Since the determination of radioactivity is the most sensitive quantification method, this technique is ideal for providing reference values to validate the use of EG‐FET technology.

The initial concentrations of the injected TMs (Note S5, Supporting Information) exceed the dynamic detection range of our EG‐FET immunosensors (Figure 3C). Therefore, dilution is necessary to bring the concentrations within the detectable range, capture complete time‐series data, and minimize sample volume requirements. In our indirect detection method, tuning of dilution factors is crucial. The signal response exhibits an inverse relationship with TM concentrations: lower signal responses are observed at earlier time points (closer to injection) when TM concentrations are higher, while higher signal responses occur at later time points as TM concentrations decrease.

Notably, the signal for scFv increases earlier than for IgG4, reaching saturation at 24 h regardless of the dilution factor (Figure S6, Supporting Information). In contrast, the signal for IgG4 remains low until the dilution factor optimally aligns with the sensor's dynamic range, even after 24 h, reflecting the differing lifetimes of scFv and IgG4 TMs. Figure 4D shows the response of EG‐FET immunosensors to serum samples collected at various time points, with the samples subjected to 10× and 100× dilutions. The “% response signal” is defined in Figure S7, Supporting Information. This figure demonstrates how signal responses vary with different dilution factors over the same time intervals. At 6 h post‐injection, significant differences in the response signals between the 10× and 100× dilutions indicate that the sensor's dynamic range is positioned within the corresponding concentration range. By analyzing multiple dilution factors, we can estimate the onset of the signal and infer TM availability in the sample.

To further explore the pharmacokinetics of these immunotherapeutic drugs, we expanded our collection of mouse serum samples. The EG‐FET sample preparation and measurement procedure are described in Note S5, Supporting Information. Due to varying TM concentrations in each serum sample, the transition of the EG‐FET signal between the “OFF” and “ON” states occurs at different dilution factor thresholds. Typically, higher concentrations result in lower signals, necessitating a higher dilution factor to achieve a sufficiently strong sensing response. We utilized the estimated dilution factor corresponding to a 50% of ON (maximal) signal of EG‐FET (ED50) (described in Figure S7, Supporting Information) as a figure of merit (FOM) for data comparison. Figures S8 and S9, Supporting Information, illustrate the procedures for obtaining the FOM for each sample. Meanwhile, the data from radioactivity measurements was obtained as described in Note S4, Supporting Information. A summary of the results from EG‐FET sensors and radioactivity measurements of scFv and IgG4 TMs in the serum of injected mice is presented in Figure 4E,F, respectively.

The estimated half‐life for scFv is 15 ± 0.8 min by EG‐FET and 12 min by radioactivity measurements. In contrast, IgG4 exhibits a longer half‐life, estimated to be almost 9 h by radioactivity measurements and 14  h by EG‐FET sensors. A comparative summary of half‐life calculations using different methods is given in Table S1, Supporting Information. Note that these cited results were obtained with a 1‐phase instead of a 2‐phase decay fitting model, as in the PET study. This discrepancy in the determination of the IgG4 TM half‐life time can be attributed mainly to the lower time resolution of the EG FET measurements, compared to that of radioactivity imaging methods. Additionally, further deviations are related to the sample size constraints. In contrast, although high‐temporal‐resolution PET measurements were anticipated to provide more data points for improved fitting and, consequently, more accurate results, these measurements may not precisely reflect serum extraction experiments due to potential variations in pharmacokinetics, limited sample size, and partial volume effect, especially in mice, leading to underestimation of blood concentration.^[^ ^50^ ^]^ While radioactivity‐based biodistribution experiments and EG‐FET measurement provide (semi)‐quantitative data for TM concentration in both blood and serum, they are limited with regard to the number of examined time points due to animal welfare aspects. It is noteworthy that radioactivity biodistribution and EG‐FET measurements in serum extraction experiments were conducted on identical mice, enabling direct comparison as illustrated in Figure 4E,F.

While our study aims to offer valuable insights into the potential application of FET‐based electronic biosensors on clinically relevant samples, we acknowledge certain limitations associated with our research methodology, such as a time delay between radioactivity and EG‐FET measurements (to reach the radioactive decay), and need to use different mice to ensure the completeness and statistical relevance of the half‐life estimates. Future research endeavors will attempt to address current limitations to improve the accuracy and reliability of our findings.

Even though the absolute estimations differ between methods, consistent results from all approaches underline the markedly shorter lifespan of scFv compared to IgG4 TMs. This observation is corroborated by PET images (Figure 4D–F) and biodistribution studies (Figure S11, Supporting Information). These results reinforce the distinct pharmacokinetic profiles of scFv and IgG4 and emphasize the potential utility of FET‐based biosensors in elucidating such critical differences in biological samples. A notable strength lies in their independence from radioactive labels in all measurements, making them cost‐effective and ready to use. Looking ahead, our study lays the groundwork for continued exploration and refinement to facilitate more comprehensive insights into pharmacokinetics. Ongoing optimization and validation efforts are essential to fully exploit the capabilities of this technique in research and clinical settings.

## 3 Conclusion and Outlook

FET‐based biosensors have long demonstrated remarkable results as diagnostic tools, and yet they are rarely employed in alternative contexts, such as therapy development and monitoring. In this proof‐of‐concept study, we explored the potential of FET‐based biosensors for immunotherapeutic drug monitoring, particularly in the UniCAR system. Leveraging the versatile nature of the UniCAR system, we have developed an innovative assay applicable to a wide range of TMs. Our indirect assay, built upon our previous EG‐FET biosensing system, exhibits exceptional sensitivity as shown for TMs targeting human FAP which we selected for proof of concept in our study.

Our key findings highlight the successful implementation of an indirect detection approach, eliminating the need for extensive system modification and biomolecular engineering. Particularly noteworthy is the almost 1000‐fold higher sensitivity of the EG‐FET biosensor compared to traditional ELISA, especially in serum samples. The achieved IC50 values, ranging from 84 fM for IgG4 TMs to 750 fM for scFv TMs, emphasize the potential of our approach for detection in the clinical setting. To further illustrate the advantages of our method, including time efficiency and other key specifications, a comparison with other techniques used in this study is provided in Table S2, Supporting Information.

Furthermore, we challenged our technique with samples from an in vivo experiment. Despite the presented limitations, our approach facilitates the construction of the pharmacokinetic profiles of two TMs. Notably, our device consistently revealed a significantly shorter lifespan (15 min) of scFv compared to the lifespan of size‐extended IgG4‐based TM (14 h), which closely aligns with the results obtained from radioactivity and PET measurements. This finding underscores the great potential of using FET‐based biosensors for pharmacokinetic studies, highlighting the technique's independence from radioactive labels.

Meanwhile, further optimization efforts are essential to unlock its potential in clinical and research applications. First, fine improvements in hardware are necessary, including efforts to minimize fabrication variations, and enhance the stability of the reference electrode, ensuring consistent and reliable sensor performance. Second, advancements in receptor immobilization techniques are crucial for improving sensor reproducibility. This involves developing reliable methods for quantifying surface coverage, refining immobilization protocols, and incorporating more sophisticated binding models, such as the ones based on McGhee and von Hippel theory,^[^ ^52^ ^]^ to improve the understanding of affinity, steric hindrance effects, and dynamics of antibody–protein interactions. In addition, the stability of immobilized layers over extended storage periods should be thoroughly investigated. These improvements will also provide quality control measures to ensure reproducibility. Lastly, validation with larger sample sizes is needed to confirm the robustness and generalizability of the findings, ensuring the system's reliability across a range of experimental conditions.

The EG‐FET configuration offers compelling advantages, such as a disposable sensing chip and a compact, cost‐effective readout system, making it highly suitable for widespread clinical adoption. Our study is a crucial step in advancing FET‐based biosensors and bioelectronics, laying the foundation for their potential application in therapeutic drug monitoring devices. As we prepare for a new era of precision medicine in immunotherapy, these innovations could hold significant promise for improving patient care and treatment outcomes in the near future.

## 4 Experimental Section

4.1

4.1.1

##### Materials

For the experiments carried out in this study, we utilized the following materials and reagents: anti‐La 5B9 monoclonal antibody (αLa 5B9 mAb) and E5B9 peptide, anti‐FAP scFv TM (α FAP scFv TM), and IgG4 TM (α FAP IgG4 TM), which were designed and produced as described previously;^[^ ^34^ ^]^ PBS solution was prepared according to product instructions from tablets (PBS, P4417, Sigma‐Aldrich), TWEEN 20 (P1379, Sigma‐Aldrich), cysteamine (Cys, M9768, Sigma‐Aldrich), glutaraldehyde (GA, G5882, Sigma‐Aldrich), BSA (A2HZDRhpiuhp153, Sigma‐Aldrich), nitric acid solution (HNO_3_, 695 041, Sigma‐Aldrich), ammonium hydroxide solution (NH_4_OH, 221 228, Sigma‐Aldrich), potassium chloride (KCl, P3911, Sigma‐Aldrich), potassium ferrocyanide trihydrate (P745.1, Carl Roth), potassium ferricyanide (7971.1, Carl Roth), ethanol (EtOH, K928.3, Carl Roth), 2‐propanol (IPA, 59 300, Sigma‐Aldrich), silver (Ag) wire (327 026, Sigma‐Aldrich); Ag/AgCl paste; platinum (Pt) wire (267 201, Sigma‐Aldrich), PDMS (Sylgard 184, Dow Corning), and sodium chloride (NaCl, S9888, Sigma‐Aldrich).

##### EG‐FET Biosensing Platform

In summary, the fabrication of the sensing chips was conducted in‐house. A 10 nm Cr layer and a 100 nm gold layer were deposited onto cleaned and patterned glass substrates to create the sensing electrode surfaces and corresponding contact pads. Individual sensing electrode assumed a 1 × 1 mm square‐shaped configuration, and each chip accommodated 32 such electrodes. An insulating PDMS reservoir was meticulously affixed to the chip to facilitate liquid handling and define the sensing surface. For potentiometric measurements, we engineered a multiplexed readout platform that operated using a single commercial FET transducer. The entire system was proficiently controlled through an Arduino Uno microcontroller board. During the operational phase, an Ag/AgCl reference electrode was immersed in the measurement liquid (0.1 mM PBS) to maintain a constant potential within the solution. The FET reader operated in constant charge mode with predefined values of the *V*_DS_ and *I*_D_ of the FET transducer.

##### EG Gold Electrodes Functionalization

The gold electrodes were electrochemically cleaned by cyclic voltammetry to attach the peptide sequences to the surface. The electrodes were incubated in cysteamine and later GA to link E5B9 peptides to the gold surface. BSA was used to block the remaining surface. The functionalized chips were stored in the refrigerator at 5 °C until the next experiment.

##### EG‐FET Biosensing Settings and Measurement

For each measurement, 0.5 mL of the diluted sample was introduced into the PDMS reservoir and allowed to interact with the sensing surface for 15 min. Subsequently, the sample was removed, and the electrode underwent three washes using PBS containing TWEEN20 (0.05% v/v). After washing, the PDMS reservoir was filled with 0.6 mL of diluted PBS (0.1 mM, pH 7.4), and the reference electrode (featuring cured Ag/AgCl paste on an Ag wire) was immersed in the solution within the reservoir. The Ag wire contact of the reference electrode was connected to the output of the buffered voltage divider to set the reference electrode potential (*V*_R_) to 2.5 V. The software was employed to establish the constant charge mode of the commercial FET (BSS138N, Infineon Technologies) with specified values *V*_DS_ = 400 mV and *I*_D_ = 100 μA. The multiplexed readout was executed across six EG electrodes by switching to the next electrode every 0.2 s. The mean value of the output signals *V*_R_ and source voltage (*V*_S_) was recorded by averaging five acquired samples corresponding to each recorded time point for each EG electrode. Data acquisition persisted for 180 s to ensure voltage signal stabilization and enough data points for averaging of the signal after the stabilization period. The initial 60 s of recorded data are typically considered necessary to establish a stable reference potential in a low ionic strength solution, such as a 100× diluted PBS solution. To ensure signal stabilization, all data presented in this study are averages of measurements recorded between 120 and 180 s.

##### Animal Models

All animal experiments were carried out according to the guidelines of the German Regulations for Animal Welfare. The protocols were approved by the local Ethical Committee for Animal Experiments (DD24.1‐5131/449/49). As described previously by us,^[^ ^34^ ^]^ female naval medical research institute (NMRI) nude mice (Rj:NMRI‐Foxn1^nu/nu^, Janvier Labs) were subcutaneously injected with 2 × 10^6^ HT1080 and 1 × 10^6^ HT1080 FAP cells in 50 μL PBS each, on the left and right hind leg, respectively. Tumor size was monitored 3 times a week by caliper measurements. Tumor‐bearing mice were included in the experiments 7–10 days post tumor cell injection, when tumors reached a volume of at least 400–700 mm^3^. For in vivo experiments with EG‐FET sensor and radioactivity measurements, mice were i.v. injected with 0.6–1.1 MBq (10–16 pmol) anti‐FAP scFv or IgG4 TM, radiolabeled as published elsewhere.^[^ ^34^ ^]^ About 1 min after tracer injection, a 100 μL blood sample was taken from anaesthetized mice by puncture of the retrobulbar venous plexus. At dedicated time points, narcotized mice were sacrificed via cardiac puncture and terminal blood collection was performed (more details in Note S4, Supporting Information). Both, blood samples from early (*T*_0_) and final time point (*T*_X_) were immediately analyzed for weight and radioactivity concentration, and used afterward to isolate the serum from whole blood. After a coagulation time of at least 30 min at room temperature, blood samples were run for 5 min at 3000 g at 4 °C. The clear supernatant was transferred to a new vial and the centrifugation process was repeated. Serum samples were measured again (weight and radioactivity concentration) and stored at −20 °C until further analysis by EG‐FET biosensor technology (Note S5, Supporting Information). In addition to blood, major organs (e.g., liver, kidney, spleen) as well as tumors were taken from the sacrificed mice and analyzed for weight and radioactivity concentration (see Figure S11, Supporting Information).

## Abbreviations

**Table:** 

EDTA	Ethylenediaminetetraacetic acid
Fc	Fragment crystallizing
His	Hexa‐histidine tag
Radio‐TLC	Radio thin‐layer chromatography
SDS–PAGE	Sodium dodecyl–sulfate polyacrylamide gel electrophoresis

## Conflict of Interest

The authors declare no conflict of interest.

## Author Contributions

**Trang‐Anh Nguyen‐Le**: data curation (lead); formal analysis (lead); investigation (lead); writing—original draft (lead); and writing—review and editing (equal). **Christin Neuber**: formal analysis (equal); investigation (equal); methodology (equal); writing—original draft (supporting); and writing—review and editing (supporting). **Isli Cela**: formal analysis (equal); investigation (equal); writing—original draft (supporting); and writing—review and editing (supporting). **Željko Janićijević**: formal analysis (supporting); methodology (supporting); and writing—review and editing (supporting). **Liliana Rodrigues Loureiro**: investigation (supporting); methodology (supporting); and writing—review and editing (supporting). **Lydia Hoffmann**: data curation (supporting) and investigation (supporting). **Anja Feldmann**: funding acquisition (supporting); methodology (equal); and supervision (equal). **Michael Bachmann**: methodology (supporting); project administration (supporting); supervision (supporting); and writing—review and editing (supporting). **Larysa Baraban**: conceptualization (lead); funding acquisition (lead); methodology (equal); supervision (lead); and writing—review and editing (equal). **Trang‐Anh Nguyen‐Le**, **Christin Neuber**, and **Isli Cela** contributed equally to this work.

## Supporting information

Supplementary Material

## Data Availability

The data that support the findings of this study are available from the corresponding author upon reasonable request.
