# Supplementary material for: Toward Personalized Immunotherapeutic Drug Monitoring with Multiplexed Extended‐Gate Field‐Effect‐Transistor Biosensors
Source: Small Sci. 2025 Feb 3;5(5):2400515. doi: 10.1002/smsc.202400515 (PMC12087767; doi:10.1002/smsc.202400515)
Supplement: Supplementary file 1 — Supplementary Material [file SMSC-5-2400515-s001.pdf]

Towards Precision CAR T-cell Immunotherapy: Extended Gate FET Biosensors for Personalized Drug Monitoring

Trang-Anh Nguyen-Le[1*], Christin Neuber[1*], Isli Cela[1], Željko Janićijević[1], Liliana Rodrigues Loureiro[1], Lydia Hoffmann[1], Anja Feldmann[1], Michael Bachmann[1], Larysa Baraban[1,2,†]

[1] Institute of Radiopharmaceutical Cancer Research, Helmholtz-Zentrum Dresden-Rossendorf e. V. (HZDR), 01328 Dresden, Germany

[2] Else Kröner Fresenius Center for Digital Health, TUD Dresden University of Technology, Dresden, Germany

[*] *These authors made equal contributions to this work*

† Corresponding author: l.baraban@hzdr.de

Note 1: ELISA experiment.

The enzyme-linked immunosorbent assay (ELISA) was performed using transparent 96-well plates (Nunc 167008, Thermo Fisher) and an Imaging Multimode Reader (Cytation5, Agilent). E5B9 peptide coating on the well plates was done at a concentration similar to that used for EG electrode functionalization (50 µg mL^{-1}, 50 µL per well). Notably, peptides were diluted in ELISA coating buffer (Cat#421701, Biolegend) rather than the PBS buffer utilized in the EG functionalization process. Peptides were allowed to adsorb to the plate overnight at 5°C, followed by five washes with PBS (10 mM, pH 7.4) containing 0.05% (v/v) TWEEN 20. The plate surface was then blocked with a 3% (w/w) skim milk powder solution for 1 hour, followed by five washes with PBS-TWEEN 20.

In the direct assay, the stock solution of αLa 5B9 monoclonal antibody (mAb) was diluted in PBS to achieve concentrations ranging from 1 ng mL^{-1} to 10 µg mL^{-1}, with 50 µL added per well. In the indirect assay, E5B9 peptide stock solution was diluted to concentrations ranging from 10 pg/mL to 100 µg/mL. It was mixed with a fixed concentration of αLa 5B9 mAb in a 1:1 volume ratio, vortexed briefly, and incubated outside of the well plate. After 30 minutes, each mixture with different E5B9 peptide concentrations was then added to the well plate (50 µL per well). In both assays, the reaction was halted after 1 hour, and the plate was washed five times with PBS-TWEEN 20.

For the colorimetric measurement, a secondary antibody containing horseradish peroxidase (HRP) was employed (Goat anti-Mouse IgG (H+L) Secondary Antibody, HRP #31430, Thermo Fisher) targeting αLa 5B9 mAb. The secondary antibody stock solution was diluted at a 1:1000 volume ratio and added to the well plate (50 µL per well). After 1 hour of incubation, the plate was thoroughly washed five times with PBS-TWEEN 20.

Colorimetric assays were conducted by adding 3,3′,5,5′-tetramethylbenzidine (TMB) substrate (BD Bioscience), prepared by mixing it with hydrogen peroxide in a 1:1 ratio, to the samples (100 µL per well). HRP-mediated oxidation of TMB led to a color change in the solution to varying shades of blue. The reaction was terminated after 3 minutes by adding H_2SO_4 (0.05 M, Acros Organics) at a volume 100 µL per well. This resulted in the solution turning yellow, and its intensity was measured via absorbance at 450 nm. The ELISA results are depicted in **Figure S5**.

Note 2: Production, modification and radiolabeling of anti-FAP scFv and IgG4 TMs

Design and cloning of lentiviral vector p6NST50 encoding for either the αFAP scFv TM or αFAP IgG4 TM alongside transduction of 3T3 cells, TM synthesis by 3T3 cells, and TM purification by Ni-NTA affinity chromatography were performed as previously described [1] . After purification, TMs were dialyzed against PBS and the concentration and purity of the TMs were determined using Sodium dodecyl-sulfate polyacrylamide gel electrophoresis (SDS-PAGE) and Western Blot as well as by measurement with NanoDrop (ThermoFisher) . Modification of αFAP TMs with the chelator NODAGA and subsequent radiolabeling with positron emitter Copper-64 was performed as described previously[1] . Radiochemical yield and radiochemical purity were analyzed by radio thin layer chromatography (radio-TLC; solid phase: iTLC-SG (Agilent), mobile phase: PBS) and radiochromatography (radio-HPLC; solid phase: Advance BioSec (Agilent), mobile phase: PBS) using a sample of the radiolabeled TMs in 2 mM aq. Ethylenediaminetetraacetic acid (EDTA) solution. Radiolabeled TMs were purified by spin filtration using Millipore Amicon®Ultra-4 centrifugal filter and PBS containing 2 mM EDTA and 0.0067% Dodecyl-β-D-maltoside (DDM). Consequently, the TMs were analyzed by HPLC and, in addition, by radio-SDS-PAGE.

Note 3: PET study

PET imaging was performed as previously described [1]. In brief, mice were i.v. injected with 10-15 MBq (60-80 pmol) radiolabeled αFAP scFv or IgG4 TM and emission data were acquired continuously for the dedicated time points (dynamic PET scan 0-60 min p.i., static PET scans 6/ 24/ 48 h p.i.). PET data were reconstructed using Mediso Tera-Tomo™ 3D iterative reconstruction including attenuation correction. Images were post-processed and analyzed using ROVER (ABX) and displayed as maximum intensity projections (MIPs) at the indicated time points and scaling. For PET data quantification, 3D volumes of interest (VOI) were created applying a fixed threshold from mask maximum (20% for tumor, heart, kidney, liver; 30% for spleen) for delineation of the organs of interest in the appropriate time frames (highest accumulation of the tracer). VOIs were transferred to all time frames for determination of standardized uptake values (SUV_{mean}) and time activity curves (TACs).

Note 4: Biodistribution study

To analyze organ distribution of αFAP TMs, mice were i.v. injected with 0.6-1.1 MBq (10-16 pmol) of radiolabeled αFAP scFv or IgG4 TM. About 1 min after tracer injection, a 100 µl blood sample was taken from narcotized mice by puncture of retrobulbar venous plexus. At dedicated time points (αFAP scFv: 5/15/30/60 min/ 3h/ 6h p.i.; αFAP IgG4: 30/60 min/ 3/6/24/48 h p.i.; each n=2) narcotized mice were sacrificed via cardiac puncture and terminal blood collection was performed. Both blood samples from early (T_0) and final time point (T_X) were immediately analyzed for weight and radioactivity concentration and, afterwards, proceeded to isolate serum from whole blood. After a coagulation time of at least 30 min at room temperature, blood samples were run for 5 min at 3000 × g at 4°C. Clear supernatant was transferred to a new vial and centrifugation process was repeated. Serum samples were stored at -20°C until analysis by EG-FET biosensor measurements. In addition to blood, major organs (e.g. liver, kidney, spleen) as well as tumors were taken from the sacrificed mice and analyzed for weight and radioactivity concentration. The radioactivity of the TM was measured before injection in the syringe in Becquerel (Bq). The recorded value was corrected for the lost amount of sample in the mouse tail during injection. Based on the molar radioactivity of the TM (scFv: 58,2 kBq/mol, IgG4: 64,8 kBq/mol) and the blood amount in each mouse (78µL/g of body weight [2]) the injected molar concentration was calculated. SUV [% Max]

values are calculated as a percentage of recorded radioactivity at T_x with respect to initial injected radioactivity t_0. Using this percentage and injected molar concentration, estimated concentrations at t_x were calculated.

Note 5: EG-FET measurement of αFAP TMs in mouse serum samples

Ex vivo measurements were conducted to assess the ability of our sensor to detect Target Modules (TMs) in samples from living organisms. Depending on body weight of the mice initial TM concentration at time of injection in serum was 7.6 ± 1.4 nM and 13.0 ± 1.6 nM for αFAP scFv and aFAP IgG4 TM, respectively. Serum samples were collected at various time points post-injection (αFAP scFv: 5/15/30/60 min/ 3h/ 6h p.i.; αFAP IgG4: 30/60 min/ 3/6/24/48 h p.i.) for analysis as described in Supplementary Note 3. For each mouse, a 1-minute p.i. blood sample and an endpoint sample were obtained. Due to volume limitations, the samples were diluted to reach the required sample volume for incubation in the reservoir (0,5 mL). To determine the most efficient dilution, we tested a range from 10 to 10^7 dilution factor. The 1-minute p.i. sample was diluted to factors of 10 and 100. All the samples were mixed in a 1:1 ratio with the αLa 5B9 mAb probe at a concentration of 1 pM (150 pg mL^{-1}) in PBS solution (10 mM, pH 7.4). To ensure homogeneous interaction of the probe with the TMs, after mixing, the samples were placed on a rotator for 15 minutes at room temperature. As a baseline, the 10x dilution of the 1-minute post-injection sample for IgG4 TM and the 100x dilution of the 1-minute post-injection sample for scFv TM was mixed in a 1:1 ratio with 1 pM αLa 5B9 mAb probe in PBS solution (10 mM, pH 7.4). After the functionalization process, the BSA solution is removed from the PDMS wells, and the chip is washed with PBS (10 mM, pH 7.4) supplemented with TWEEN 20 (0.05% v/v). After repeating the washing step three times, the chip is additionally washed with PBS (10 mM, pH 7.4) to remove any TWEEN 20 residue. The chip is then incubated in PBS (10 mM, pH 7.4) for 5 minutes, and afterward, a measurement is performed as described in the EG-FET biosensing measurement section. This measurement provides an initial reference to the system's signal response. Subsequently, we incubated 0.5 mL of each sample for 15 minutes, starting from the baseline to the highest dilution factor, followed by washing and measuring in diluted PBS (0.1 mM, pH 7.4).

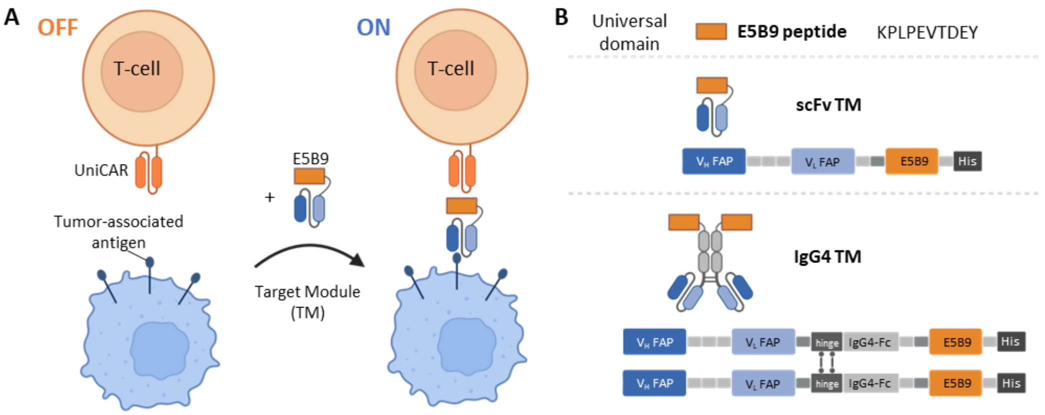

Figure S1: Schematic representation of UniCAR system in cancer immunotherapy and particularly in the targeting of FAP. (A) UniCAR T-cells consist of an extracellular single-chain fragment variable (scFv) directed to the peptide

epitope E5B9. By adding an intermediary target module (TM) consisting of a scFv that recognizes tumor-associated antigens and the epitope E5B9 that interacts with UniCARs, redirecting UniCAR T-cells to attack tumor cells is made possible. **(B)** Construction of different molecules involved in this study, including the amino acids sequence of the E5B9 epitope as well as the structure of scFv-based TM and IgG4-based TM, the latter bearing hinge region and Fc domain of the human IgG4. V_H: variable domain of the antibody heavy chain, V_L: variable domain of the antibody light chain, Fc: fragment crystallizing, His: hexa-histidine tag.

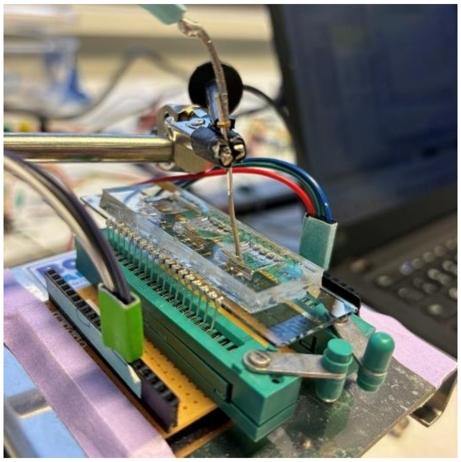

Figure S2: Image of our potentiometric EG-FET sensing system during operation

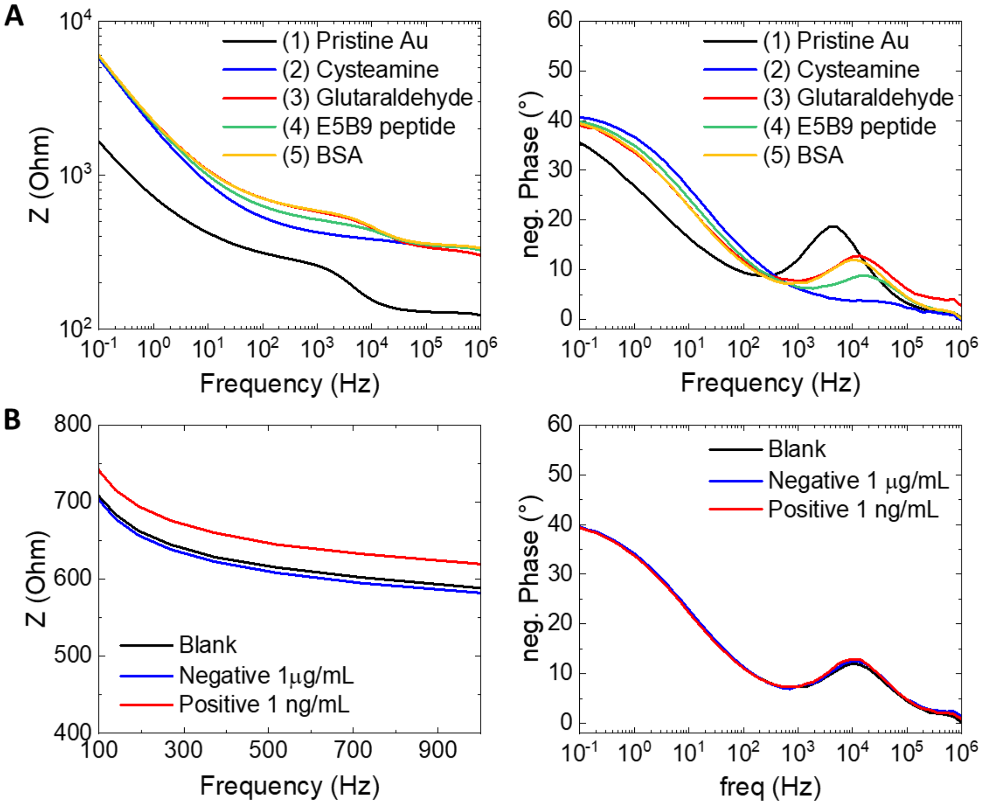

Figure S3: In situ monitoring of sensing electrode functionalization process using electrochemical impedance spectroscopy (EIS). **(A)** Bode plot of impedance and phase shift after each functionalization step **(B)** Bode plot of impedance and phase shift after incubation with negative control sample (mouse IgG antibody at 1 µg mL^{-1}) and positive control sample (αLa 5B9 mAb at 1 ng mL^{-1})

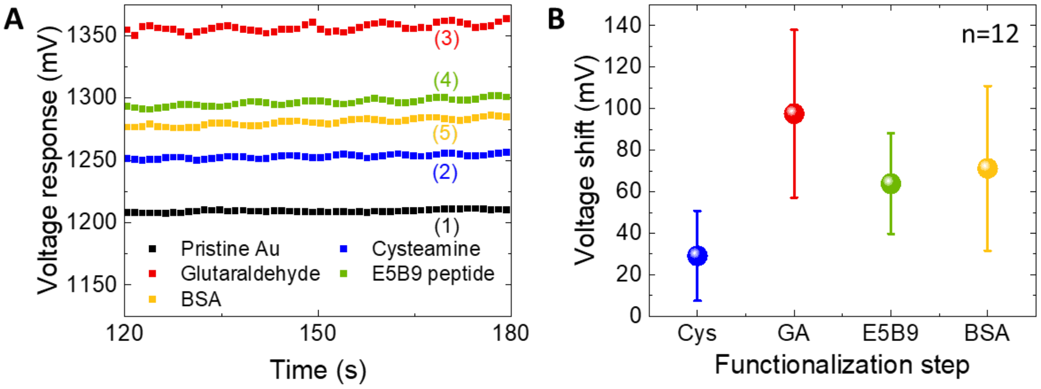

Figure S4: In situ monitoring of sensing electrode functionalization process using our potentiometric EG-FET sensing system **(A)** Recorded real-time response after each functionalization step **(B)** Average voltage shift compared to pristine gold after each functionalization step for 12 electrodes.

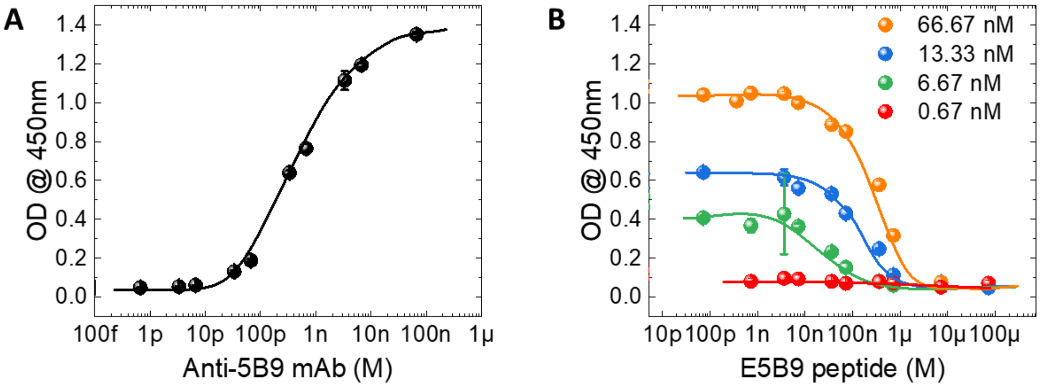

Figure S5: ELISA calibration results for **(A)** probe molecules (αLa 5B9 mAb) and **(B)** indirect assay for E5B9 peptides with different probe concentrations.

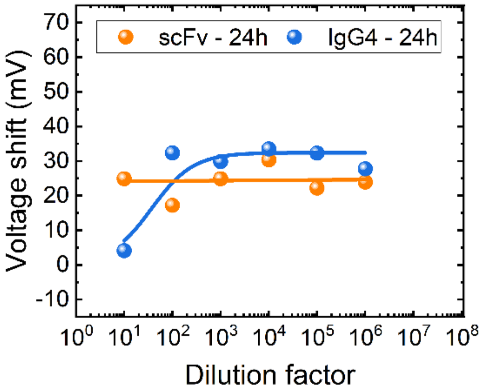

Figure S6: EG-FET measurement results of serum mice sample 24 hours post injection of αFAP scFv and IgG4 TM.

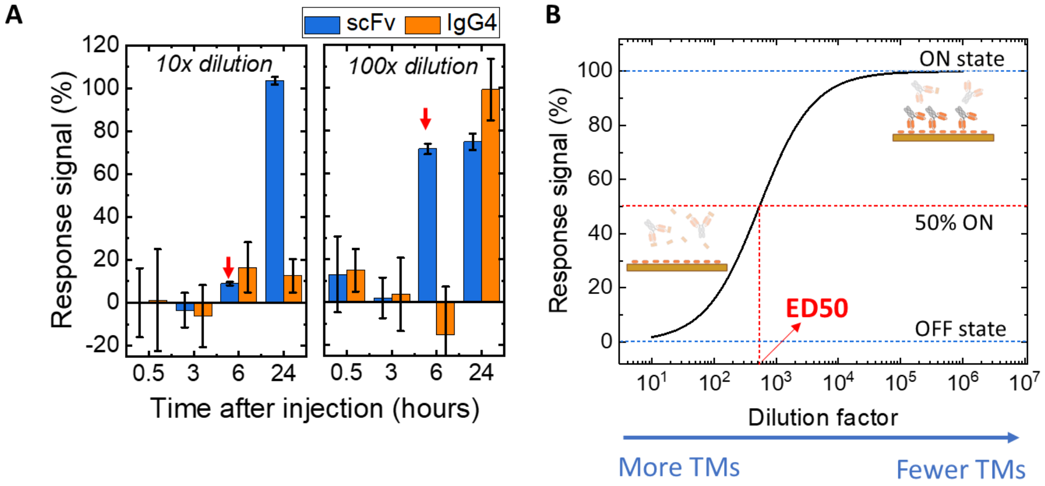

Figure S7: **(A)** EG-FET signal response to sample at different time points and dilution factors. Red arrows indicate the time point when the signal starts to shift (Figure 4D from the maintext); **(B)** Illustration of effective dilution factor for a half-maximal signal of each serum sample containing TMs in an indirect measurement approach.

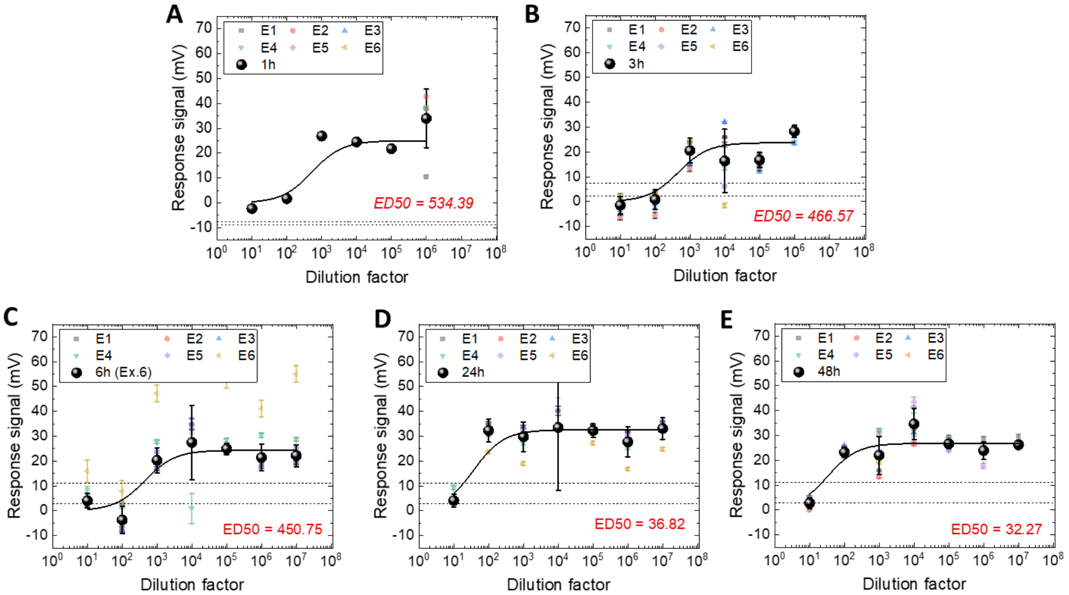

Figure S8: EG-FET measurement results of serum mice sample **at** 1 hour **(A)**, 3 hours **(B)**, 6 hours **(C)**, 24 hours **(D)** and 48 hours **(E)** after injection of IgG4 TM.

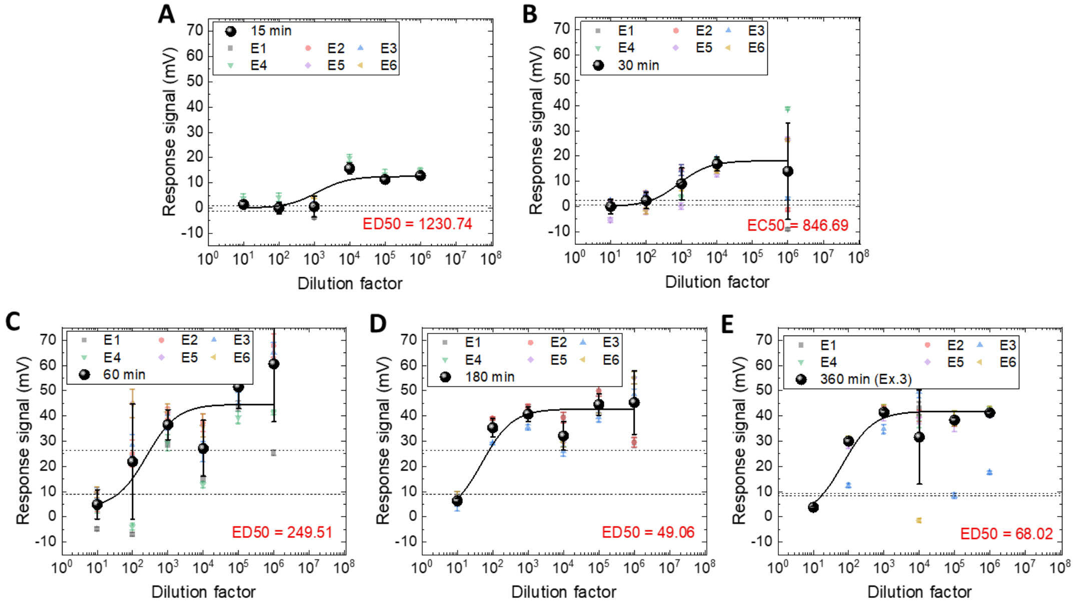

Figure S9: EG-FET measurement results of serum mice sample at 15 minutes **(A)**, 30 minutes **(B)**, 60 minutes **(C)**, 180 minutes **(D)** and 360 minutes **(E)** after injection of scFv TM.

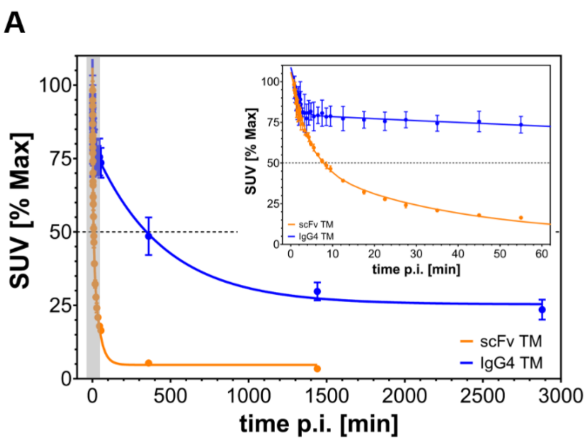

B

PET data	scFv TM	IgG4 TM
fit model	2 Phase*	2 Phase*
half-life$_{fast}$	3.1 min	64.4 min
half-life$_{slow}$	32 min	36 h
% fast phase	56	38
R^2	0.9952	0.8236
half-life$_{total}$	11 min	16 h

* plateau set to 0

Figure S10: PET measurement of scFv and IgG4 TM in tumor-bearing mice (n≥3). After reaching maximum SUV, time-activity curves **(A)** for scFv and IgG4 TM in heart, representing blood pool, were fitted by two-phase exponential decay (plateau set to 0) to obtaine blood half-life of TMs **(B)**.

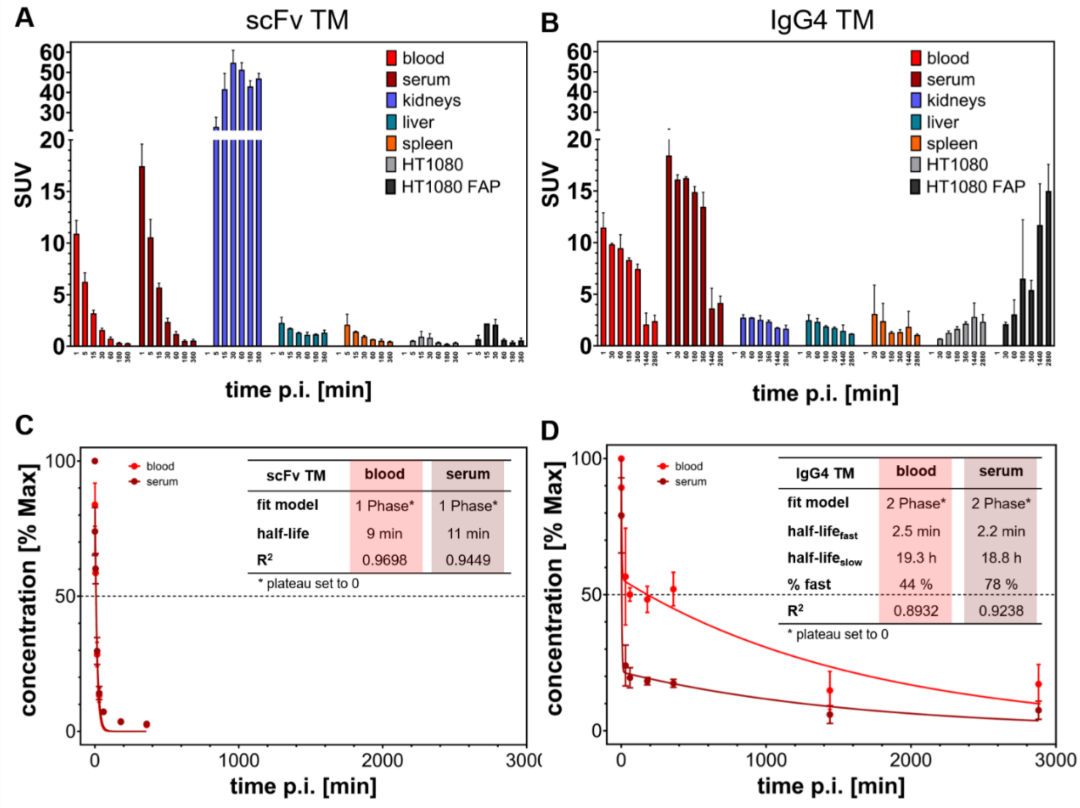

scFv TM	blood	serum
fit model	1 Phase*	1 Phase*
half-life	9 min	11 min
R^2	0.9698	0.9449

* plateau set to 0

IgG4 TM	blood	serum
fit model	2 Phase*	2 Phase*
half-life$_{fast}$	2.5 min	2.2 min
half-life$_{slow}$	19.3 h	18.8 h
% fast	44 %	78 %
R^2	0.8932	0.9238

* plateau set to 0

Figure S11: Biodistribution and pharmacokinetic results from radioactivity measurement of TMs in tumor-bearing mice. To investigate activity concentration in different organs and tissues of interest, mice were sacrificed at specific time points after i.v. injected of radiolabeled anti-FAP TMs (scFv TM: 5, 15, 30, 60 minutes, 3 , 6 hours p.i.; IgG4 TM: 30, 60 minutes, 3, 6, 24, 48 hours p.i.). Organs of interest were removed and radioactivity concentration (delineated as SUV) was measured.

Table S1: Comparative analysis of pharmacokinetics of TMs in different approaches

	αFAP scFv TM				αFAP IgG4 TM			
	PET	RA (Blood) Biodistribution	RA (Serum) Biodistribution	EG-FET	PET	RA (Blood) Biodistribution	RA (Serum) Biodistribution	EG-FET
**Fit model**	2-phase	1-phase	1-phase	1-phase	2-phase	2-phase	1-phase	1-phase
**R^2**	0.997	0.974	0.968	0.998	0.862	0.897	0.773	0.731
**Half-life**	Fast: 3.1 min<br>Slow: 32 min<br>Total: 11.0 min	9.0 min	12.0 min	15.4 min	Fast: 64.4 min<br>Slow: 36.0 h<br>Total: 16.0 h	Fast: 2.5 min<br>Slow: 19.3 h	8.7 h	14.2 h

**Highlighted cells are values reported in the main text*

Table S2: Comparison of key specifications across analytical methods used in this study. Note that the comparison assumes the use of pre-coated ELISA plates and EG-FET chips (no bio-immobilization time included).

	**PET**	**Radioactivity measurement**	**ELISA**	**Our EG-FET**
**Detection method**	Radioactivity	Radioactivity	Optical	Electrical
**Labeling** *(*)*	Before administration<br>~5-7 hours *(**)*	Before administration<br>~5-7 hours *(**)*	During measurement<br>~45 minutes	No
**Preparation time** *(***)*	5 minutes	5 minutes	1.5 – 2 hours	15 minutes
**Measurement time**	Real-time *(****)*	1-5 minutes	1-5 minutes	1-5 minutes
**Multiplexing**	Imaging method	1 *(with an option for automatic sampling)*	96 wells	32 electrodes/3 wells
**Sample volume**	N/A	>1 mL	50-100 µL/well	25-50 µL/well
**Detection limit**	10^{-15} to 10^{-12}M<br>(depending on isotope and system)	10^{-15} to 10^{-12}M<br>(depending on isotope and system)	10^{-10}M	10-^{15}M

(*) Refer to the time effort for labeling the TMs (analytes)

(**) Refer to the method for modification of αFAP TMs with NODAGA and radiolabeling with copper-64 [1]

(***) Refer to the time and effort required for sample preparation and incubation, from a ready-to-use serum sample to its placement in the device detector.

(****) Reconstruction time varies from a few minutes to hours depending on the number of time points collected.

References:

(1) Loureiro, L. R.; Hoffmann, L.; Neuber, C.; Rupp, L.; Arndt, C.; Kegler, A.; Kubeil, M.; Hagemeyer, C. E.; Stephan, H.; Schmitz, M.; Feldmann, A.; Bachmann, M. Immunotheranostic Target Modules for Imaging and Navigation of UniCAR T-Cells to Strike FAP-Expressing Cells and the Tumor Microenvironment. *Journal of Experimental & Clinical Cancer Research* **2023**, *42* (1), 341. https://doi.org/10.1186/s13046-023-02912-w.

(2) O'Connell, K.; Mikkola, A.; Stepanek, A.; Vernet, A.; Hall, C.; Sun, C.; Yildirim, E.; Staropoli, J.; Lee, J.; Brown, D. Practical Murine Hematopathology: A Comparative Review and Implications for Research. *Comp Med* **2015**, *65*, 96–113.
